# Advances in Medicine

**Published:** 1877-03

**Authors:** 


### Editorial.

### ADVANCES IN MEDICINE.

We glean the following important items from reports of committees made to the Medical Society of Virginia, as found in the Transactions published in the *Virginia Medical Monthly* for January, 1877:

"*Dilute Phosphoric Acid,*" prepared from glacial phosphoric acid, as ascertained by Dohme & Remington, contains soda; and even when phosphorus is used in the preparation, nearly three per cent. of arsenic is found, unless freed from this impurity by sulphuretted hydrogen. The test of purity, as reported, is the failure to throw down a precipitate when tincture of the chloride of iron is added to the dilute phosphoric acid.

*Explosive Prescriptions.*—The reporters mention as explosive mixtures, permanganate of potassium 10 parts, with 15 parts each of alcohol and water; fluid extract of uva ursi with sweet spirit of nitre; and the mixture of chromic acid with glycerine.

*Physostigma.*—Without having experimented with the remedy, we have thought proper, from reports of its effects, to classify calabar bean with excito-motor sedatives, and advise its use in the treatment of tetanus, (Westmoreland's Acology, page 329,) and are pleased to find the opinion corroborated by the report of committee on "advances in therapeutics." We copy the entire report on this remedy *verbatim*, as follows:

"The therapeutical employment of the calabar bean is the rational outcome of its physiological action as a spinal depressent. Dr. Dickerson and Mr. Pollock have recently reported three cases of tetanus successfully treated with physostigma (*Lancet*, Sept. 2, 1876). The reporter is enabled to speak from clinical observation of the therapeutical value of the drug in tetanus. In a case of traumatic origin, in which the symptoms were exceedingly threatening from the outset, the calabar bean, largely employed, seemed to the reporter to have fairly accomplished the successful result. In recording the case, the reporter indulged in the following reflections: From the close observation of the action of the calabar bean, he is persuaded that its beneficial effects were manifested in allaying the irritability of the peripheral nerves, and in blunting at the same time the central nervous system in the receptivity of painful impressions, thus blotting out the salient pathological factors of the disease (*Virginia Medical Monthly*, Jan., 1876)."

*Jaborandi.*—In the report of the committee on "advances in therapeutics," we find several interesting and instructive items. A very satisfactory account is given of jaborandi, from the reports of various experimenters. By vivisection and otherwise, it has been positively determined that this drug possesses very useful medicinal virtues. Until recently its definite properties were not known; and, although used and recommended in the treatment of tuberculosis for several years past, it seems to have been a kind of empirical prescription, without any definite idea of its physiological action. By occular demonstration it has been proven that the liver, pancreas, kidneys, and salivary glands are excited to increased secretion of their respective fluids by the elective action of this remedy. Having been introduced into the circulation, it was ascertained, by canulas inserted in the ducts of these glands, that the secretions were notably increased. With this information, the beneficial use of jaborandi in consumption is easy of explanation. It is known that oily substances are indispensable in the treatment of tuberculosis, and that, in order to their usefulness, they must be properly digested. The physiological fact that emulsification of fats by the pancreatic fluid must be had before they can be appropriated, is equally well established. Now, for the purpose of insuring the preparation of cod-liver oil, butter, and other fatty substances, we readily see the propriety of using jaborandi for the treatment of conditions of the human system requiring these calorifacients.

No pancreatic stimulant has, hitherto, been generally known, and we hail the discovery of this as an important addition to the list of medicinal agents whose physiological action has beeen clearly demonstrated. In cases having deficient pancreatic secretion, requiring the constant use of pancreatin, in the absence of any pancreatic stimulant the jaborandi becomes an appropriate remedy, and particularly so in view of its hepatic stimulation giving an increase of bile.

No mention is made, in the report referred to, of any influence exerted upon the stomach, increasing the quantity of gastric fluid; but since all other juices prominently connected with the process of digestion are increased by it, including that of saliva, may we not expect that this action may be also discovered? If so, the manufacturers of pepsin, pancreatin, and blue mass will find a diminution of their trade, while dyspeptics will rejoice in the use of a less expensive means of promoting digestion.

Antagonism between atropia and jaborandi was also discovered by the experimenters from whom the committee quotes. It was ascertained that after action of jaborandi had been had, the renal and hepatic secretions were diminished, and the pancreatic entirely arrested by atropia injected into a vein.

From this report we also learn that jaborandi is the Indian name given to the leaves of *pilocarpus pinnatus*, and that several preparations have been made of the drug. The active principle consists of an alkaloid called *pilocarpin*, one grain of which affords the desired effects of the remedy. The leaves in substance are given in the dose of twenty grains, and the fluid extract, half a fluid drachm.

We give these particulars in regard to the drug, hoping that much benefit may result from the introduction of this promising new remedy.

